# McKeown esophagectomy for a thoracic esophageal carcinoma patient who has a history of definitive chemoradiotherapy for esophageal carcinoma and total pharyngolaryngectomy for hypopharyngeal cancer

**DOI:** 10.1186/s12957-023-02999-7

**Published:** 2023-03-27

**Authors:** Kotaro Sugawara, Takashi Fukuda, Yutaka Kishimoto, Daiji Oka, Satoru Shirakura, Hiroaki Kanda, Yoshiyuki Kawashima

**Affiliations:** 1 Department of Gastroenterological Surgery, Saitama Cancer Center Hospital, Saitama, 362-0806 Japan; 2 Division of Head and Neck Surgery, Saitama Cancer Center, Saitama, Japan; 3 Department of Pathology, Saitama Cancer Center, Saitama, Japan

**Keywords:** Esophageal carcinoma, Total pharyngolaryngectomy, Salvage esophagectomy, Thoracoscopic surgery

## Abstract

A 64-year-old man, who had previously undergone definitive chemoradiotherapy (dCRT) and endoscopic resections for metachronous multiple esophageal squamous cell carcinoma (ESCC) and had also received total pharyngolaryngectomy (TPL) for hypopharyngeal cancer, was diagnosed with ESCC in the middle thoracic esophagus (cT3N0M0). Thoracoscopic McKeown esophagectomy was performed for the patient. Although the tumor was tightly adherent to the thoracic duct and both main bronchi, they were successfully mobilized. In order to maintain the blood supply to the trachea, we preserved the bilateral bronchial arteries and avoided prophylactic upper mediastinal lymph node dissection. Cervical end-to-side anastomosis between the jejunum and a gastric conduit was performed. Minor pneumothorax was managed conservatively, and the patient was discharged 44 days after the surgery. Overall, thoracoscopic McKeown esophagectomy was safely performed in a patient with a history of TPL and dCRT. Surgeons should be very careful to prevent tracheobronchial ischemia by optimizing the extent of lymph node dissection.

**Supplementary Information:**

The online version contains supplementary material available at 10.1186/s12957-023-02999-7.

## Background

Esophageal squamous cell carcinoma (ESCC) and head and neck cancers (HNCs) frequently occur synchronously or metachronously [1]. Esophagectomy is technically challenging in patients who have a previous history of total pharyngolaryngectomy (TPL) with free jejunal graft reconstruction for HNCs. Surgeons must be very careful when dissecting the adhesions around the cervical jejunoesophageal anastomosis. Furthermore, the extent of lymph node (LN) dissection has to be carefully considered, with the aim of preserving the blood supply to the trachea [2].

Salvage esophagectomy (SALV) after dCRT not only is technically difficult due to radiation-induced fibrosis but also is a highly invasive procedure with a high incidence of critical postoperative complications such as anastomotic leakage and tracheal necrosis. A previous study demonstrated short-term outcomes of patients undergoing concurrent TPL and esophagectomy [3], and a recent study suggested Ivor–Lewis esophagectomy to be feasible for patients who have a history of TPL [2]. Herein, we document a rare ESCC case who underwent McKeown esophagectomy after dCRT for ESCC and TPL with free jejunal graft reconstruction.

## Case presentation

In April 2008, a 51-year-old man underwent endoscopic mucosal resection for superficial ESCC (pT1b, lymphovascular invasion +) and subsequent chemoradiotherapy (50 Gy and 2 cycles of cisplatin + 5-fluorouracil regimen). The whole thoracic esophageal irradiation with elective nodal irradiation was performed. In April 2009, he was treated with TPL, free jejunal graft reconstruction, and tracheostomy for hypopharyngeal cancer. He then received multiple curative ESDs for superficial ESCC (2016, 2018, and 2020).

**Fig. 1:**
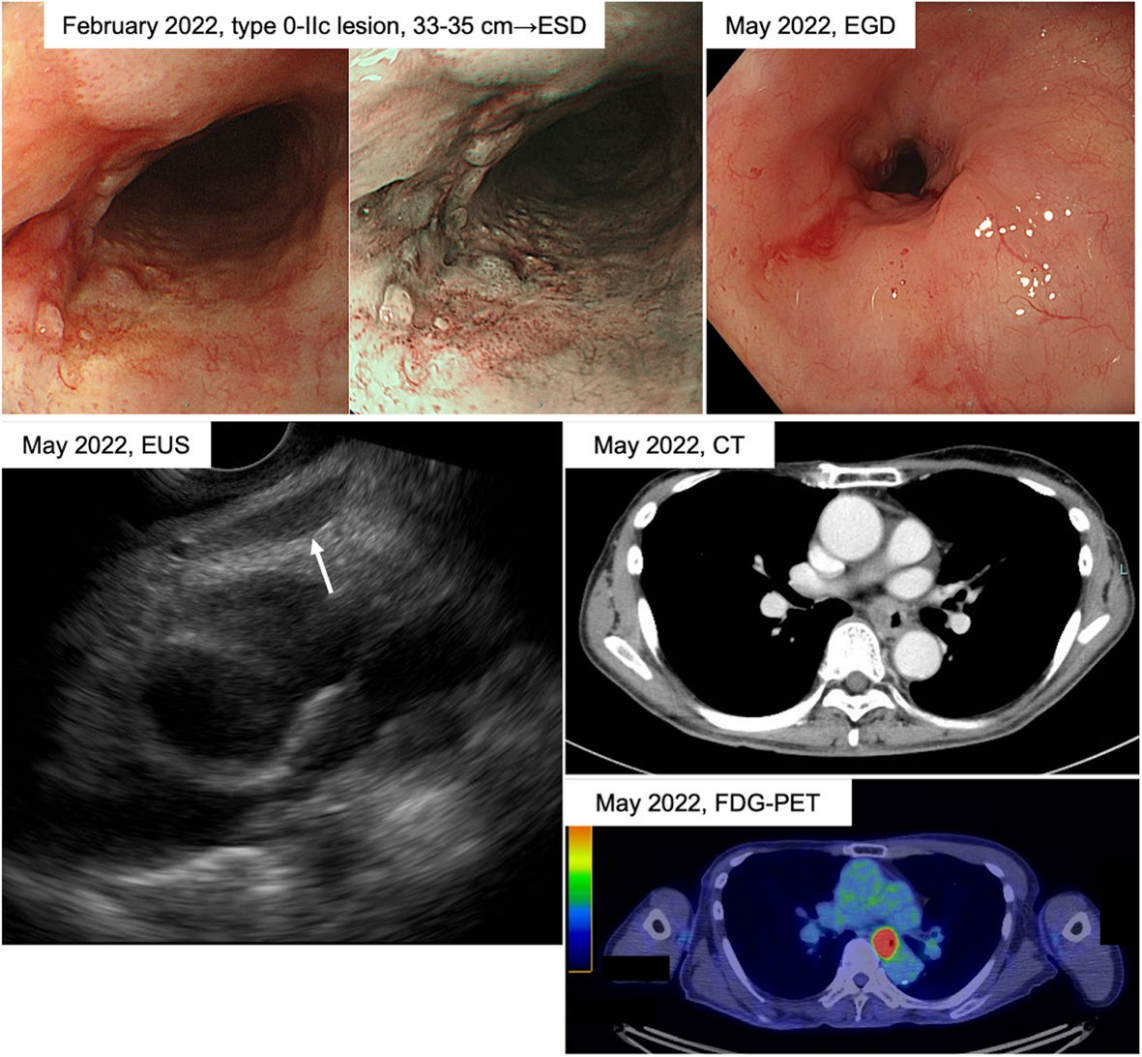
Endoscopic, CT, and FDG-PET findings before surgery. In February 2022, upper gastrointestinal endoscopy detected a type 0–2c lesion located 33–35 cm from the incisors, and ESD was performed for removal of this lesion. In May 2022, EGD and EUS detected stenosis attributable to intramural tumor recurrence in the previously treated area. CT and FDG-PET imaging detected neither LN nor distant metastases. ESD, endoscopic submucosal dissection; EGD, esophagogastroduodenoscopy; EUS, endoscopic ultrasound sonography; CT, computed tomography; FDG-PET, 18F-fluorodeoxyglucose positron emission tomography

In February 2022 (at the age of 64), he was diagnosed with thoracic superficial esophageal cancer. A type 0–2c lesion located on the left side of the wall in the middle thoracic esophagus (33–35 cm from the incisors) newly appeared (Fig. 1). Computed tomography detected neither LN involvement nor distant metastases. The tumor was classified as clinical stage 1 (cT1aN0M0), and endoscopic submucosal dissection (ESD) was performed for removal of this lesion. Curative resection had been clinically achieved; however, pathological diagnosis of tumor depth and the resection margin was very difficult since tight scar tissue had formed around the tumor. In the ESD-resected specimen, squamous cell carcinoma was detected in size of 20 × 7 mm. No vessel invasion was identified. The possibility of proper muscular layer invasion (pT2) (Supplementary Fig. 1a) and resection margin positivity (Supplementary Fig. 1b) could not be deniable. After thorough discussion in the cancer board, close observation was selected considering that the risks associated with esophagectomy were high due to the patient’s complicated treatment history.

A follow-up endoscopic examination 2 months later (May 2022) detected stenosis attributable to intramural tumor recurrence in the previously treated area (Fig. 1). Endoscopic ultrasound-guided fine needle aspiration confirmed the presence of ESCC (Fig. 1). The patient was diagnosed with middle intrathoracic ESCC, classified as clinical stage 2 (cT3N0M0) based on computed tomography and 18F-fluorodeoxyglucose positron emission tomography evaluations (Fig. 1). The artery and vein of the free jejunal graft were anastomosed with the left transverse cervical artery and the left internal jugular vein, respectively. Neoadjuvant chemotherapy was not given because the significance of readministering drugs used in the first-line therapy and combination therapy for these patients has not been established [4].

**Fig. 2:**
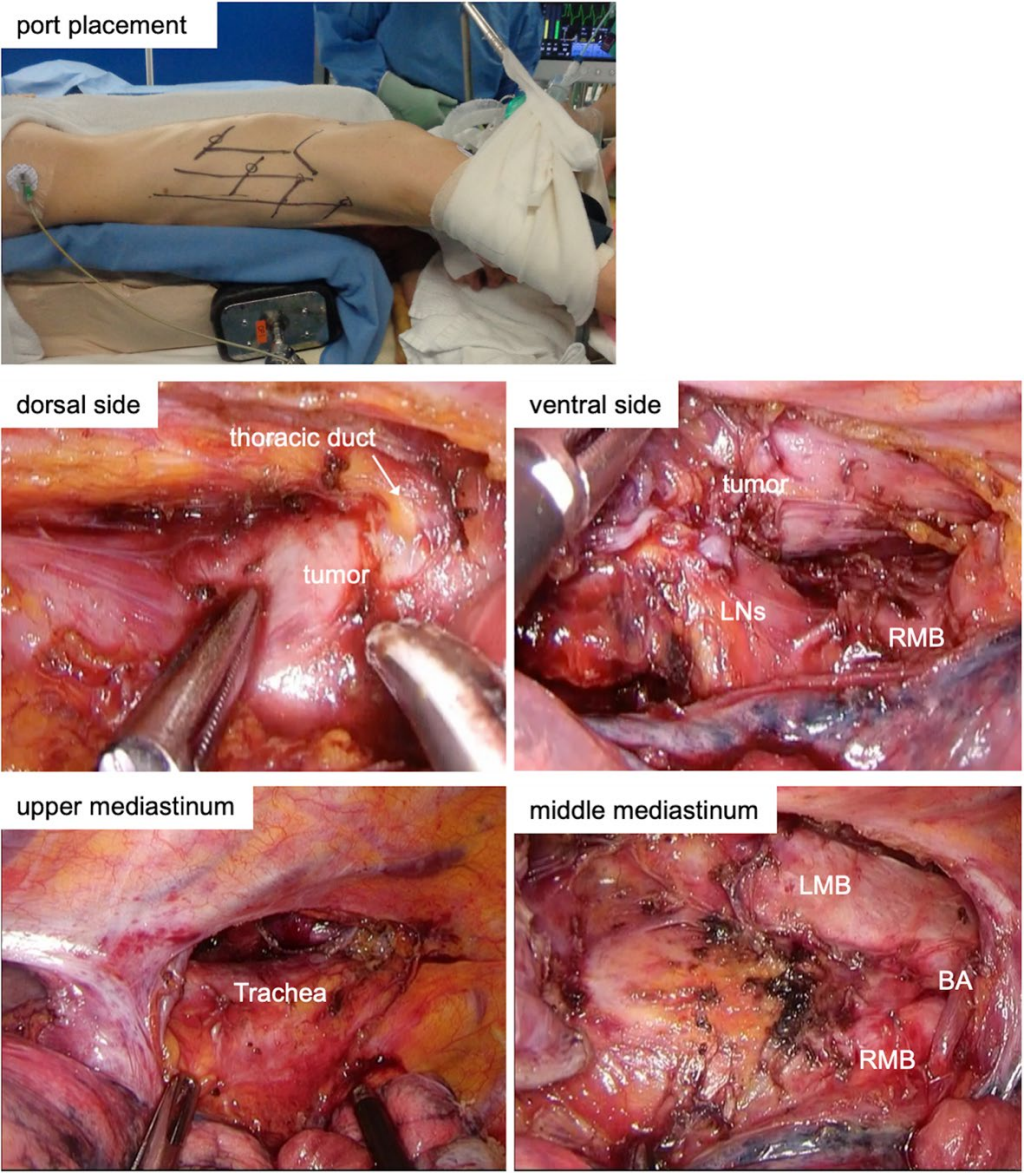
Intraoperative thoracoscopic findings. Five ports were placed, as shown. The esophagus was adherent to the thoracic duct layer with fibrosis. The ventral side of the tumor and paraesophageal and subcarinal LNs were tightly adherent to the left and right main bronchi. Prophylactic upper mediastinal lymphadenectomy was minimized. Both bronchial arteries, pulmonary branches of the bilateral vagus nerves, and the azygos arch were all carefully preserved. LN, lymph node; RMB, right main bronchus; LMB, left main bronchus; BA, brachial artery

With the patient in a prone position, the operative thoracic approach was performed by video-assisted thoracoscopic surgery with five access ports, as presented in Fig. 2. For esophagectomy after dCRT, we performed limited LN dissection, i.e., harvesting only LNs that were swollen or suspected of harboring a recurrence [5]. Although the esophagus was adherent to the thoracic duct layer with fibrosis, it was possible to preserve the thoracic duct. Although the ventral side of the tumor, paraesophageal nodes (no. 108) and subcarinal nodes (no. 107 and no. 109), also tightly adhered to the left and right main bronchi with severe fibrosis (Fig. 2), these structures were sufficiently mobilized. Prophylactic upper mediastinal lymphadenectomy around the remnant esophagus was minimized to avoid impairing the blood supply to the trachea. Both bronchial arteries, pulmonary branches of the bilateral vagus nerves and the azygos arch, were all carefully preserved (Fig. 2). Overall, only paraesophageal LNs (no. 105) were harvested in the upper mediastinum, and middle and inferior mediastinal LNs were totally removed (no. 107, no. 108, no. 109, no. 110, no. 111, and no. 112).

**Fig. 3:**
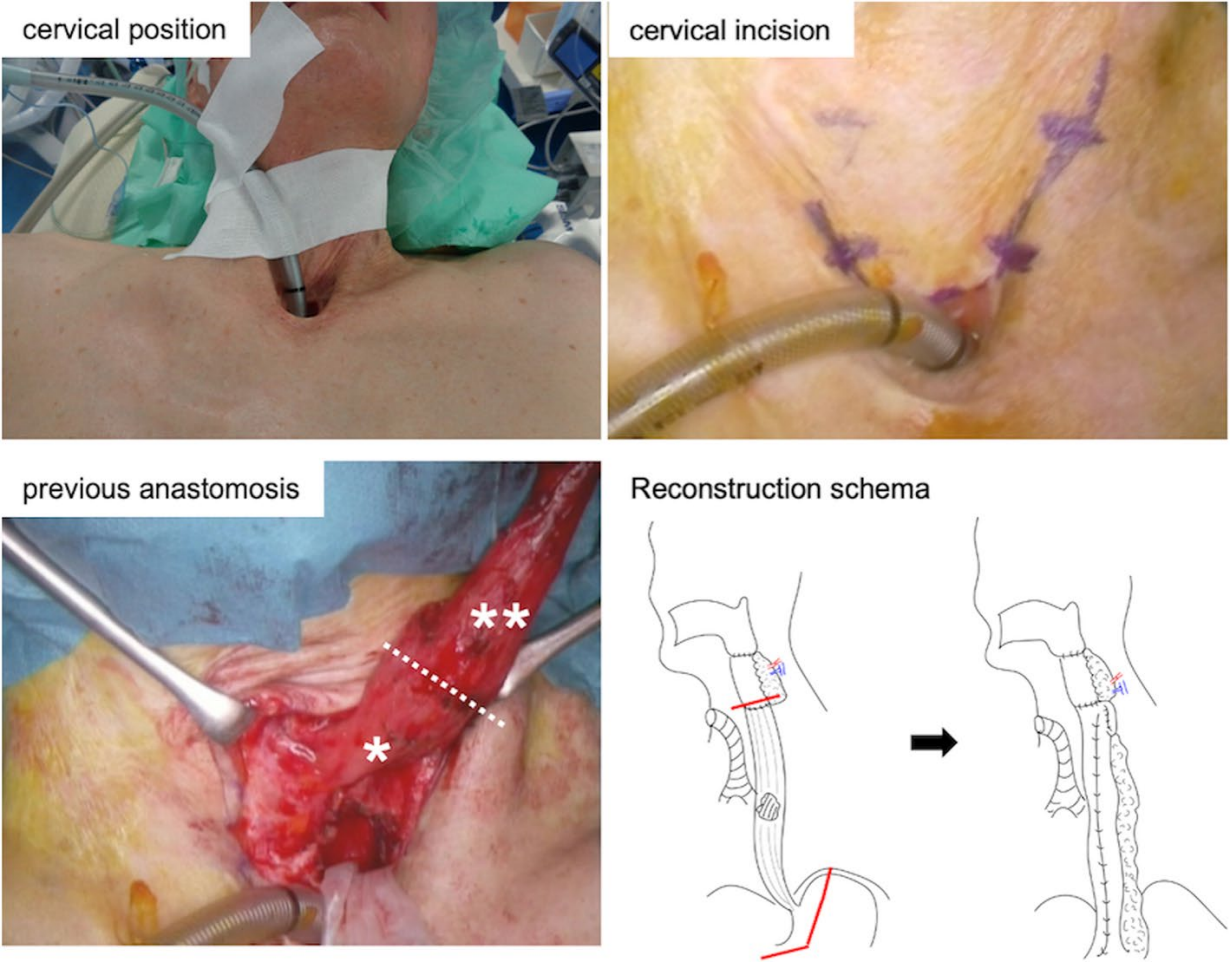
Cervical maneuver and reconstruction schema. The endotracheal tube was fixed to the cervical skin. A cervical incision was made along the upper side of the tracheostomy. The free jejunal graft and remnant esophagus were carefully mobilized. The white dotted line represents the resection line. *Jejunal graft, **esophagus. Schema showing the surgical procedure before and after esophagectomy

Next, in the supine position, a cervical incision was made along the upper side of the tracheostomy, and the free jejunal graft and remnant esophagus were then carefully mobilized (Fig. 3). The adhesion around the free-jejunal graft and adhesiolysis between the jejunoesophageal anastomosis and the membranous portion of the trachea were relatively loose. A gastric conduit was created via laparotomy and raised via the posterior mediastinal route. The esophagus was entirely removed, and end-to-side anastomosis between the jejunal graft and gastric conduit was performed (Fig. 3). The operating time was 455 min, and the estimated blood loss was 332 g.

Experienced head and neck surgeons confirmed the color tone of tracheostomy to be good after surgery. Four days after the operation, chyle pleural effusion was recognized. We changed enteral nutrition via the jejunostomy from a control enteral diet including lipid (E-7II; Clinico, Tokyo, Japan) to one without lipid (Erental; Ajinomoto Pharma, Tokyo, Japan), and chyle pleural effusion disappeared. Furthermore, minor pneumothorax was noted 7 days postoperatively and was treated by drainage with a thoracic catheter. The patient was discharged on postoperative day 44.

**Fig. 4:**
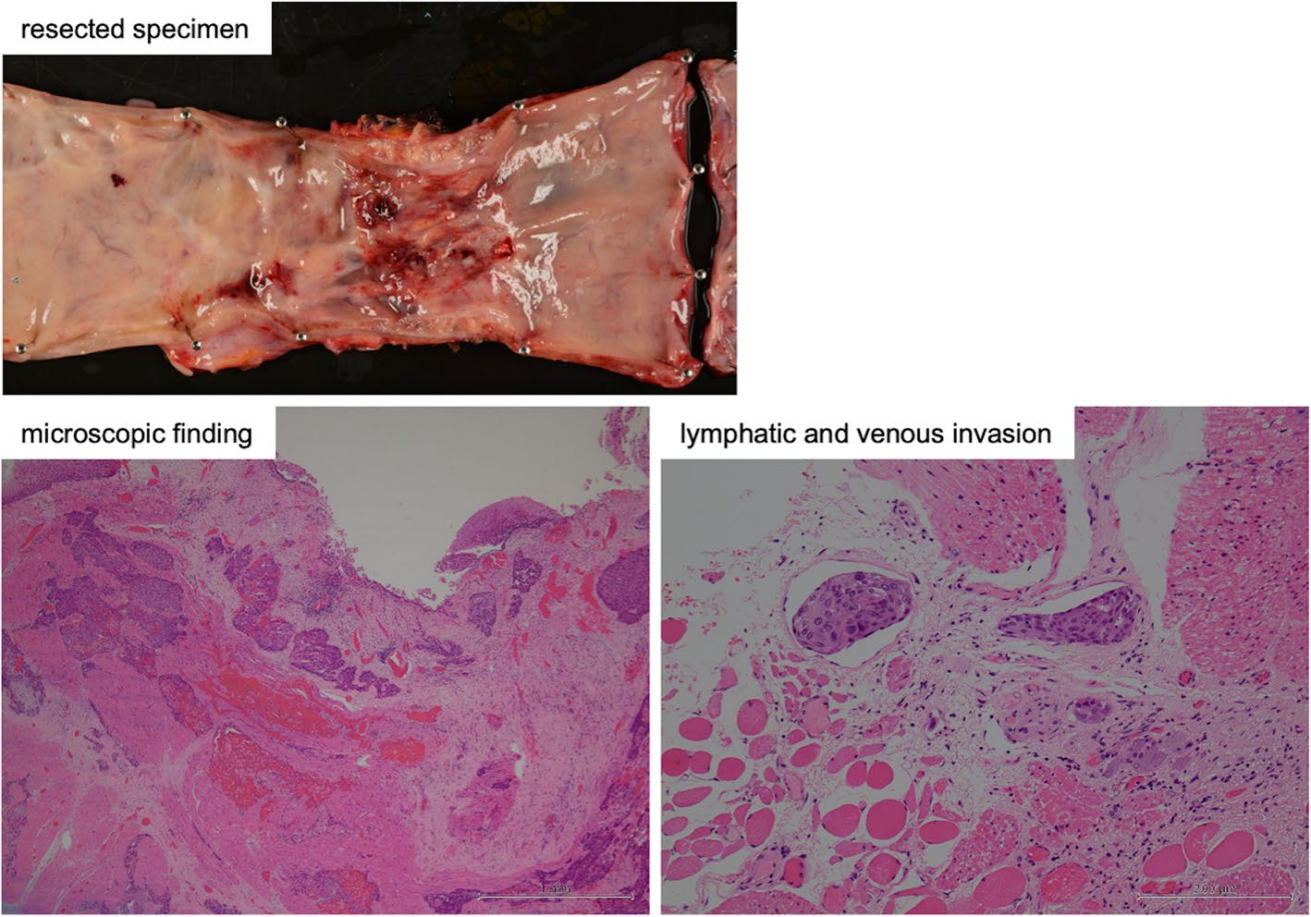
Macro- and microscopic findings of the resected specimen. Moderately differentiated squamous cell carcinoma was widely invaded in size of 155 × 30 mm beyond the proper muscular layer (pT3). Marked vessel invasion (ly3, v3) was found

The macro and microscopic findings of the resected specimen were shown in Fig. 4. Moderately differentiated squamous cell carcinoma was widely invaded in size of 155 × 30 mm beyond the proper muscular layer (pT3). Marked vessel invasion (ly3, v3) was found. Surgical margin was free (pPM0, PDM0, pRM0). The effect of preoperational therapy was slight (grade 1a). Lymph node metastases were detected in middle mediastinum LNs (no. 107, no. 109) and perigastric LNs (no. 1, no. 3, and no. 7). Pathological examination revealed the tumor to be pT3N2M0 stage 3B.

## Discussion

To our knowledge, this is the first case report describing a patient who underwent thracoscopic McKeown esophagectomy after dCRT for EC and TPL with free jejunal graft reconstruction. In such cases, surgeons should pay particularly close attention to preserving the blood supply to the trachea by carefully dissecting radiation-induced fibrosis surrounding the tumor and optimizing the extent of lymphadenectomy. Although long-term outcomes have yet to be addressed, our present patient’s course suggests McKeown thoracoscopic esophagectomy to be feasible as a salvage treatment after dCRT for ESCC patients with a previous history of TPL.

When we perform McKeown esophagectomy in a patient with a history of TPL, we must dissect adhesiolysis between the jejunoesophageal anastomosis and the membranous portion of the trachea, which increases the risk of tracheal or enteric injury [2]. Most importantly, esophagectomy after dCRT and TPL requires extra surgical devices to avoid tracheal ischemia. In fact, TPL with total esophagectomy reportedly increases the risk of postoperative fatal tracheal necrosis [6]. Therefore, we strive to preserve the bilateral bronchial arteries. Furthermore, we did not perform prophylactic upper mediastinal LN dissection in the middle and upper mediastinum in order to maintain important longitudinal tracheobronchial blood flow.

A recent study revealed that patients without clinical LN metastases rarely developed recurrence in the subcarinal LNs after salvage esophagectomy even when prophylactic upper mediastinal lymphadenectomy was not performed [5]. Therefore, our therapeutic strategy of selective lymphadenectomy is potentially reasonable for such patients without clinically positive LNs in whom it is important to maintain longitudinal tracheobronchial blood flow to avoid tracheal ischemia. It is noteworthy that the diagnostic accuracy of LN positivity remains low, especially in patients receiving radiotherapy, even when multiple diagnostic modalities are used [7].

In our present case, esophagectomy was performed long after TPL had been carried out. Previous studies have suggested tracheal blood flow bypass to potentially form within a short period (approximately 1 month) after TPL [6]. In fact, tracheal necrosis did not occur in patients undergoing staged TPL and total esophagectomy even when radical mediastinal LN dissection was performed [8]. Our results, taken together with prior findings [2, 6, 8], suggest that the interval between the two procedures is crucial for avoiding tracheal necrosis.

Esophagectomy after dCRT is technically demanding due to the impacts of high radiation doses, which produce tissue fibrosis and scarring, leading loss of clarity among the layers to be dissected. Thoracoscopic esophagectomy allows surgeons to recognize the optimal layer for dissection and microanatomy, such that this approach is also useful for salvage cases [9]. Furthermore, minimally invasive procedure is reportedly associated with favorable short-term outcomes, especially regarding postoperative pneumonia [10]. On the other hand, the safety, the oncological radicality, and the long-term survival benefits of thoracoscopic esophagectomy have yet to be fully confirmed in locally advanced EC. A randomized phase 3 trial of thoracoscopic versus open esophagectomy for thoracic esophageal cancer is now underway in Japan and is expected to establish the value of thoracoscopic esophagectomy [11].

## Conclusion

McKeown esophagectomy was safely performed in a patient with a history of dCRT for EC and TPL for hypopharyngeal cancer. Careful maneuvering and the optimization of LN dissection are crucial since CRT and TPL can cause severe adhesions and fibrosis, which in turn impair the blood supply to the trachea.

## Supplementary Information

**Additional file 1: Supplementary Fig. 1.** ESD-resected specimen. The possibility of proper muscular layer invasion (pT2) and resection margin positivity was suspected.

## Data Availability

Not applicable.

## Abbreviations

TPL: Total pharyngolaryngectomy
dCRT: Definitive chemoradiotherapy
ESCC: Esophageal squamous cell carcinoma
HNC: Head and neck cancer
LN: Lymph node
SALV: Salvage esophagectomy
EGD: Esophagogastroduodenoscopy
ESD: Endoscopic submucosal dissection
CT: Computed tomography
LVI: Lymphovascular invasion
C-D: Clavien-Dindo
