# Supplementary figures and images for: McKeown esophagectomy for a thoracic esophageal carcinoma patient who has a history of definitive chemoradiotherapy for esophageal carcinoma and total pharyngolaryngectomy for hypopharyngeal cancer
Source: World J Surg Oncol. 2023 Mar 27;21:107. doi: 10.1186/s12957-023-02999-7 (PMC10041727; doi:10.1186/s12957-023-02999-7)

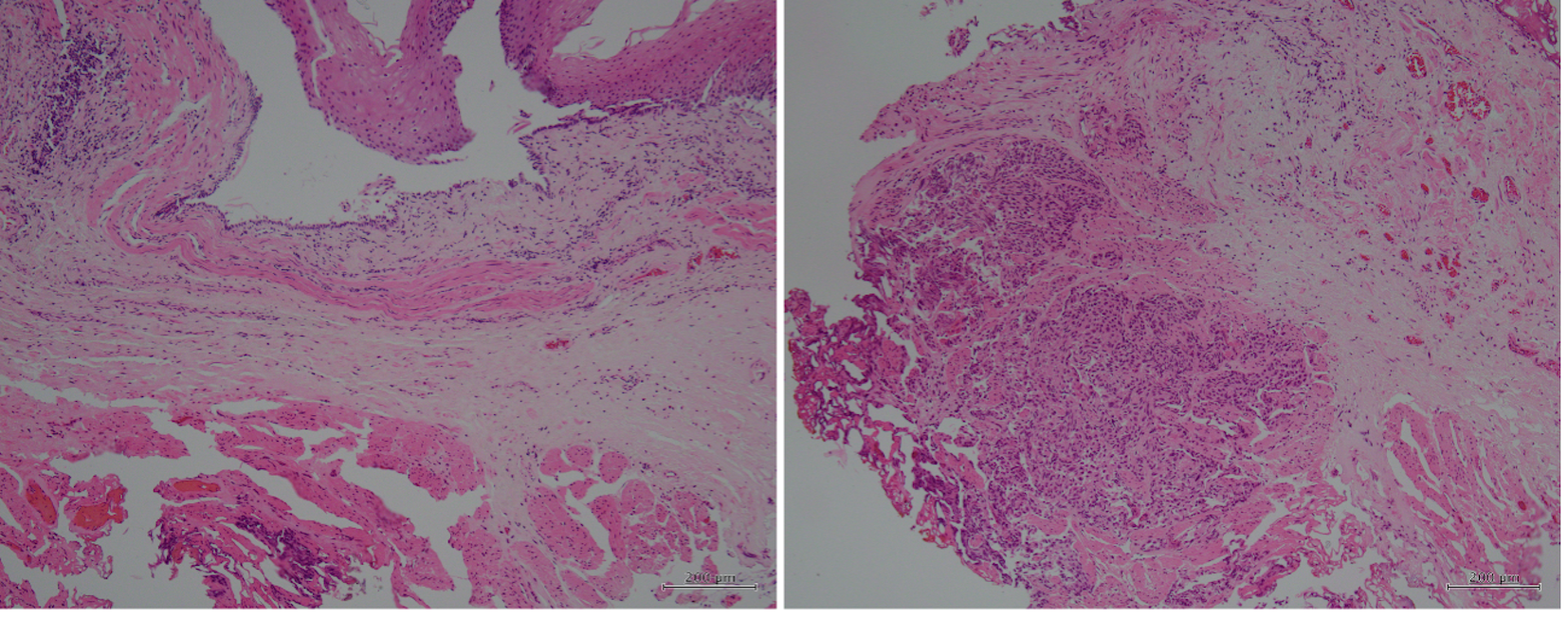

Supplement: Supplementary file 1 — Additional file 1: Supplementary Fig. 1. ESD-resected specimen. The possibility of proper muscular layer invasion (pT2) and resection margin positivity was suspected. [file 12957_2023_2999_MOESM1_ESM.tiff]
